# Association of persistent oxidative stress markers with coronary artery abnormalities in Kawasaki disease patients on follow-up

**DOI:** 10.1186/s12969-026-01249-w

**Published:** 2026-09-04

**Authors:** Rajni Kumrah, Taru Goyal, Manphool Singhal, Prateek Bhatia, Vignesh Pandiarajan, Ankur Kumar Jindal, Rakesh Kumar Pilania, Deepti Suri, Surjit Singh, Amit Rawat

**Affiliations:** 1 Allergy Immunology Unit, Department of Pediatrics, Advanced Pediatrics Centre, Post Graduate Institute of Medical Education and Research (PGIMER), Chandigarh, 160012 India; 2 Department of Radiodiagnosis and Imaging, Post Graduate Institute of Medical Education and Research, Chandigarh, 160012 India; 3 Pediatric Hematology Oncology Unit, Department of Pediatrics, Advanced Pediatrics Centre, Post Graduate Institute of Medical Education and Research (PGIMER), Chandigarh, 160012 India; 4 Division of Basic and Clinical Immunology, Department of Medicine, University of California, Irvine, USA

**Keywords:** Kawasaki disease, Endothelial dysfunction, Coronary artery aneurysms, Oxidative stress, Reactive oxygen species, Inflammation

## Abstract

**Background:**

Kawasaki disease (KD) is associated with long-term vascular sequelae, including persistent endothelial abnormalities. Data on laboratory-based markers of oxidative stress during long-term follow-up of KD patients from developing countries are limited. This study aimed to evaluate extracellular nitric oxide metabolites and neutrophil-derived reactive oxygen species (ROS) in patients with KD during follow-up and to explore their associations with coronary artery abnormalities (CAAs).

**Methods:**

In this prospective, single-centre study, 62 children with KD and 20 age- and gender-matched healthy controls (HC) were enrolled. KD patients were stratified into three groups based on time since diagnosis and further categorised by the presence or absence of CAAs. Serum nitrite and nitrate levels were measured as indicators of extracellular nitric oxide metabolism. Neutrophil ROS production was assessed using a dihydrorhodamine-123 flow cytometric assay.

**Results:**

Serum nitrite levels were comparable across the 3 groups (Group 1: 3.46 ± 1.64; Group 2: 5.04 ± 2.55; Group 3: 5.22 ± 3.00) and HC (3.33 ± 1.98) (*p* = 0.06). The serum nitrate levels in KD patients across all three groups (Group 1: 63.07 ± 41.32; Group 2: 58.63 ± 27.12; Group 3: 64.57 ± 26.36) were also comparable to HC (57.49 ± 8.68) (*p* = 0.43). In subgroup analysis, serum nitrate levels and serum nitrite levels in KD patients with CAAs and without CAAs were comparable (Group 1: *p* = 0.60; Group 2: *p* > 0.99; Group 3: *p* = 0.10) and (Group 1: *p* > 0.99; Group 2: *p* > 0.99; Group 3: *p* = 0.90), respectively. Neutrophil ROS production (ΔMFI) was increased in KD patients, with a significant rise observed in the intermediate follow-up group 2 (> 1.5–3 years) compared with healthy controls (*p* = 0.03). No significant correlations were found between oxidative stress markers and systemic inflammatory parameters.

**Conclusion:**

Children with KD exhibit persistent oxidative stress during follow-up, as evidenced by increased neutrophil ROS production, indicating ongoing cellular oxidative activity. Elevated ROS may serve as an accessible biomarker for CAAs and long-term cardiovascular risk in KD patients on follow-up.

**Supplementary Information:**

The online version contains supplementary material available at https://doi.org/10.1186/s12969-026-01249-w.

## Background

Kawasaki disease (KD) is the most common childhood vasculitis disorder and the most important cause of acquired heart disease in children in developed countries [1]. The exact cause of KD remains unknown despite extensive research, and no pathognomonic laboratory tests are available for its diagnosis [2, 3]. Cardiovascular complications are a major concern in KD patients during the acute stage. Although patients recover well if treated early, it can lead to severe cardiovascular complications in 25% of patients with KD if not diagnosed in time and even in 5–10% of treated patients.

The endothelium plays an important role in vascular homeostasis. Endothelial dysfunction (ED) is one of the earliest manifestations of atherosclerosis, often preceding other cardiovascular pathologies. ED is recognised as a key component in the pathogenesis of KD, contributing to the development of CAAs. Systemic ED may persist for many years even after resolution of signs and symptoms in patients with KD with no detectable CAAs [4].

Oxidative stress arises from excess reactive oxygen species generated during inflammation. Immune cells produce ROS, including nitric oxide (NO), leading to vascular damage, vasculitis, and progression to ED [5, 6]. Thus, the development of ED may be attributed to an imbalance between the generation and availability of ROS and NO, which can be triggered by a variety of inflammatory stimuli and oxidative stress. Under physiological conditions, shear stress from blood flow stimulates NO production, maintaining vascular homeostasis; however, reduced NO bioavailability, along with increased endothelin-1 (ET-1) levels, disrupts endothelial integrity [7].

Clinical studies have evaluated endothelial dysfunction in KD, with many reporting echocardiographic evidence of CAAs [8–12]. However, limited data are available on laboratory-based markers of endothelial dysfunction during long-term follow-up. This gap is particularly evident in developing countries, where resource constraints and inconsistent surveillance limit the availability of long-term data. This is clinically relevant, as subclinical vascular changes may remain undetected and progress to future cardiovascular complications. In this context, the present study aimed to assess inflammation-induced persistent oxidative stress markers as potential biomarkers of coronary artery abnormalities in patients with KD undergoing long-term follow-up.

## Methods

### Study design

The study was a single-centre prospective study carried out in the Allergy and Immunology Unit, Department of Pediatrics, Advanced Pediatrics Centre, Postgraduate Institute of Medical Education and Research, Chandigarh, India. The Institute Ethics Committee approved the study protocol (INT/IEC/2018/001367). Written informed consent was obtained from the parents or guardians of all participants before enrolment. Clinical details were recorded using a structured proforma, including relevant history and physical examination findings.

### Study subjects

Children with a confirmed diagnosis of KD who attended the Pediatric Rheumatology Clinic at Advanced Pediatrics Centre, PGIMER, Chandigarh, and were undergoing follow-up were included. Diagnosis had been established according to the American Heart Association (AHA) 2004/2017 criteria [1, 13].

### Subject selection

**Fig. 1:**
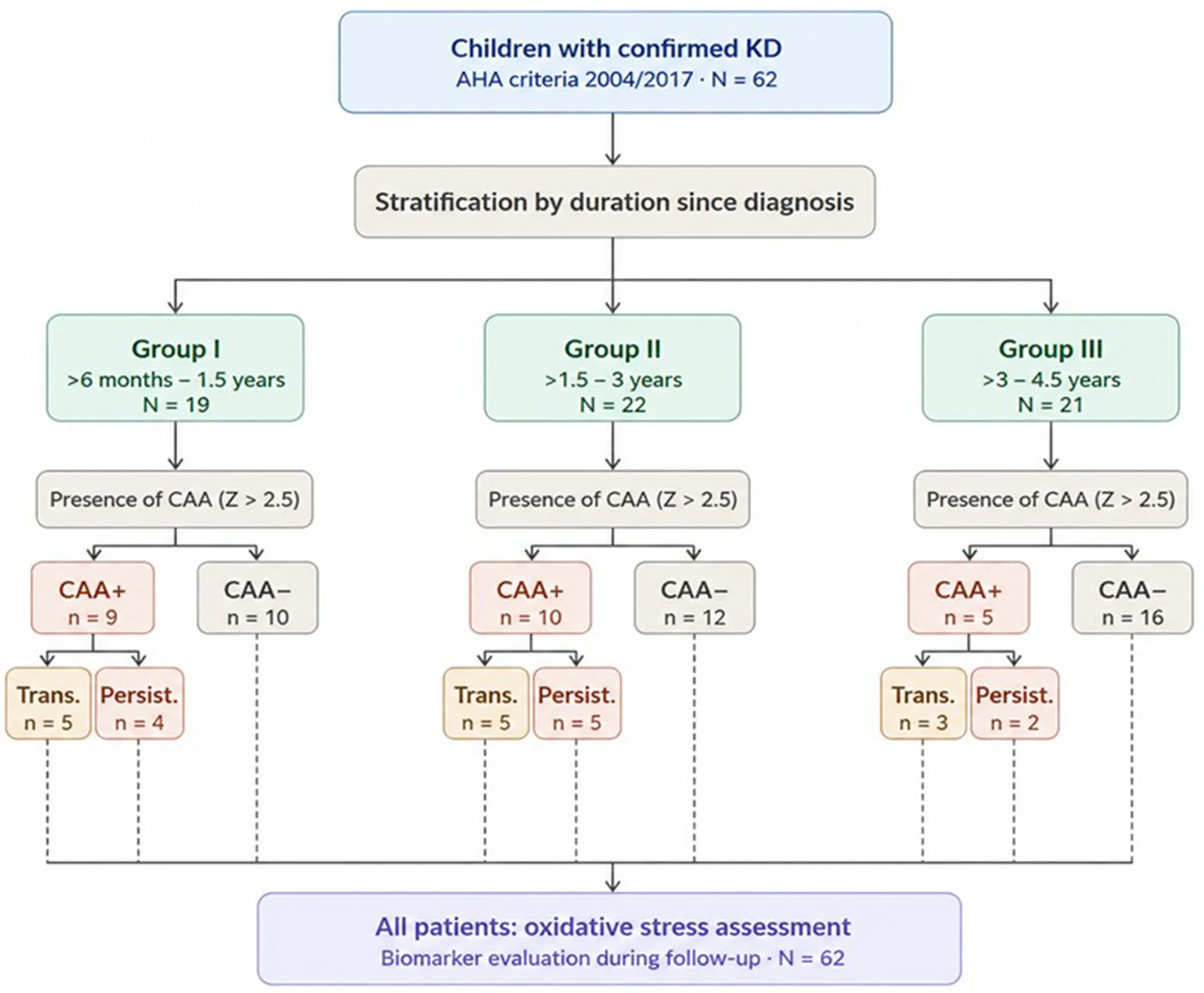
Schematic representation of the study design and patient stratification. Children diagnosed with Kawasaki disease (KD) according to the American Heart Association (AHA) 2004/2017 criteria were prospectively enrolled. Patients were stratified into three groups based on the duration of follow-up from diagnosis (> 6 months–1.5 years, > 1.5–3 years, and > 3–4.5 years). Within each follow-up group, patients were further classified according to the presence or absence of coronary artery abnormalities (CAAs) based on coronary artery Z-scores. Patients with CAAs were sub-classified as having transient or persistent aneurysms in accordance with AHA 2017 guidelines. All enrolled patients underwent assessment of oxidative stress markers during follow-up

Sixty-two (62) children with KD under follow-up were enrolled in this study. Participants were stratified into 3 groups based on the duration since diagnosis: >6 months to 1.5 years, > 1.5 to 3 years, and > 3 to 4.5 years. Participants were further categorised based on the presence or absence of CAAs [14]. Patients with CAAs were subsequently classified as having transient or persistent aneurysms according to AHA 2017 criteria, as illustrated in the workflow diagram (Fig. 1) [13].

**Fig. 2:**
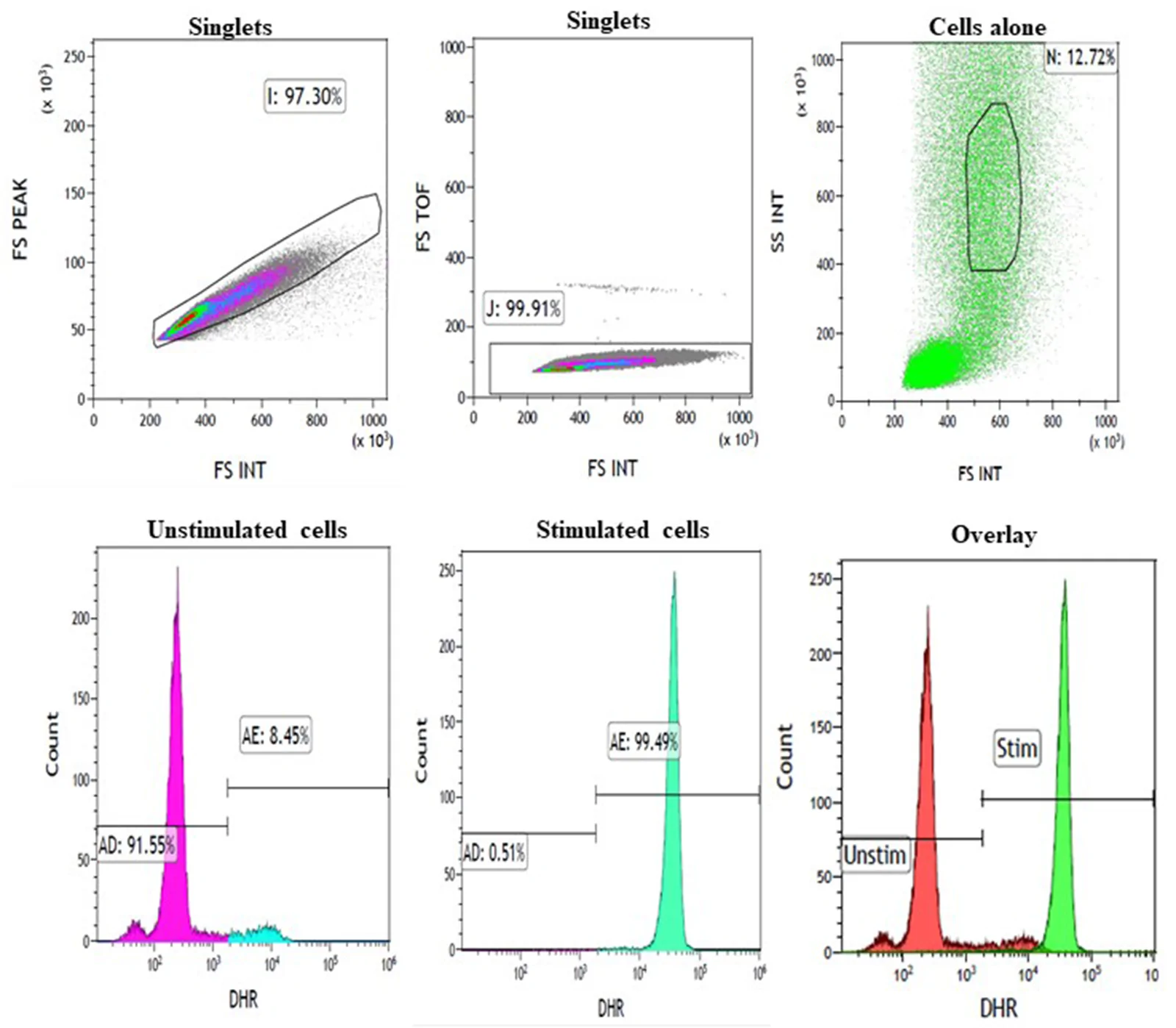
Gating strategy for data acquisition for ROS estimation. First and second graphs as singlets to exclude the doublet population in forward scatter (FS) INT vs. FS Peak and FS Time of flight (TOF) dot plots. The third graph is a dot plot (FS INT vs. SS INT), and the three major peripheral blood cell populations are segregated on the basis of size and granularity, in which FS measures size, and side scatter (SS) measures granularity of the cells. The bottom cell population is lymphocytes, the middle population is monocytes, and the top, largest population is neutrophils. Neutrophils (the largest and most granular) were gated on FSC vs. SSC. The fourth graph is a histogram of DHR vs. count for unstimulated cells, showing autofluorescence in the FITC filter. The fifth plot shows the histogram of DHR vs. count for stimulated cells, measuring the dye’s fluorescence after reduction by ROS produced by PMA stimulation

**Fig. 3:**
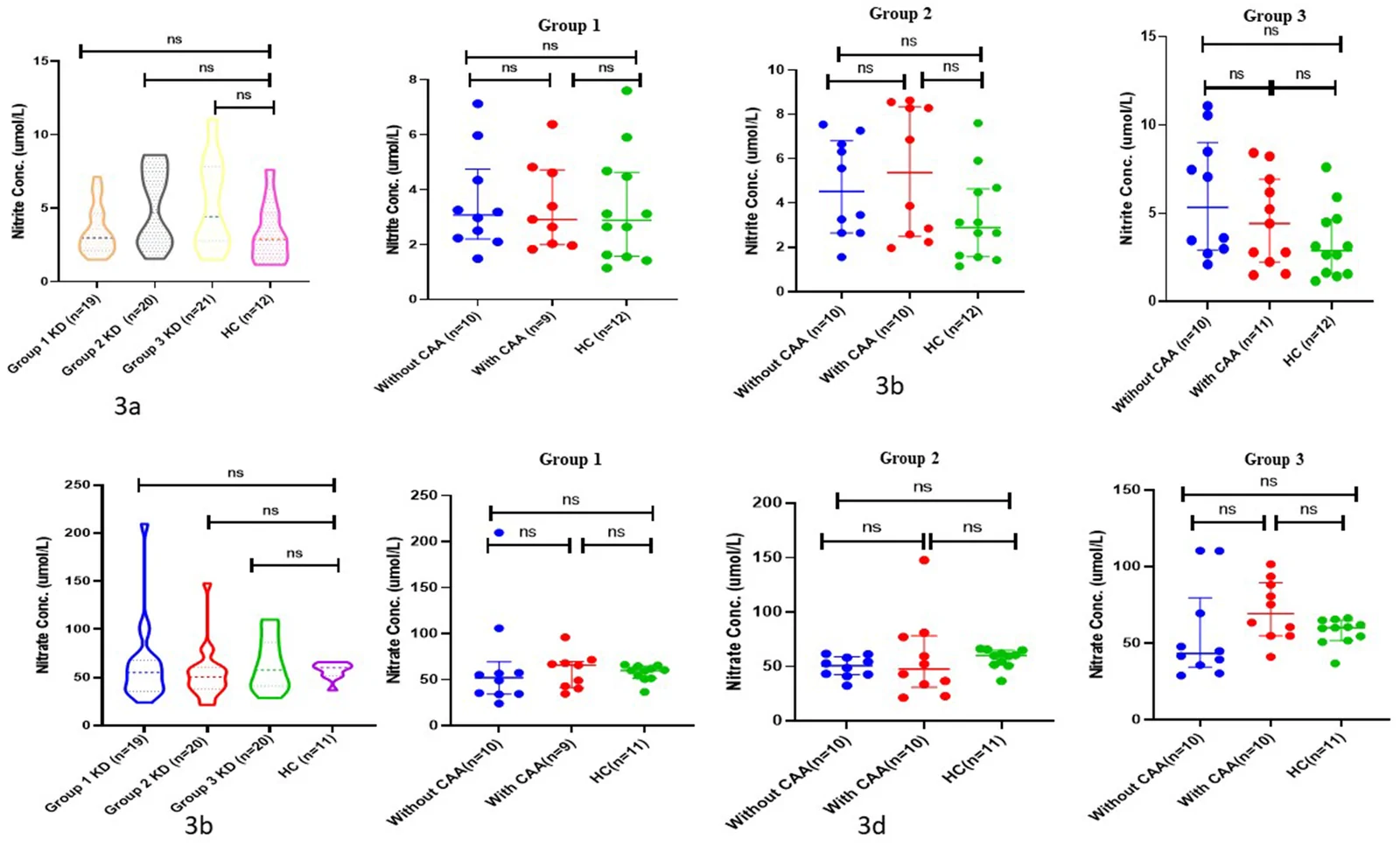
Comparison of serum nitrite and nitrate levels in patients with KD and healthy controls. (**a**) Comparison of nitrite levels between KD patients across different study groups and healthy controls; (**b**) Subgroup analysis comparing nitrite levels in KD patients with CAAs and HC; (**c**) Comparison of nitrate levels between KD patients across different study groups and healthy controls; (**d**) Subgroup analysis comparing nitrate levels in KD patients with CAAs and HC. Abbreviations: KD-Kawasaki disease, HC-healthy control, CAAs-coronary artery aneurysms

**Fig. 4:**
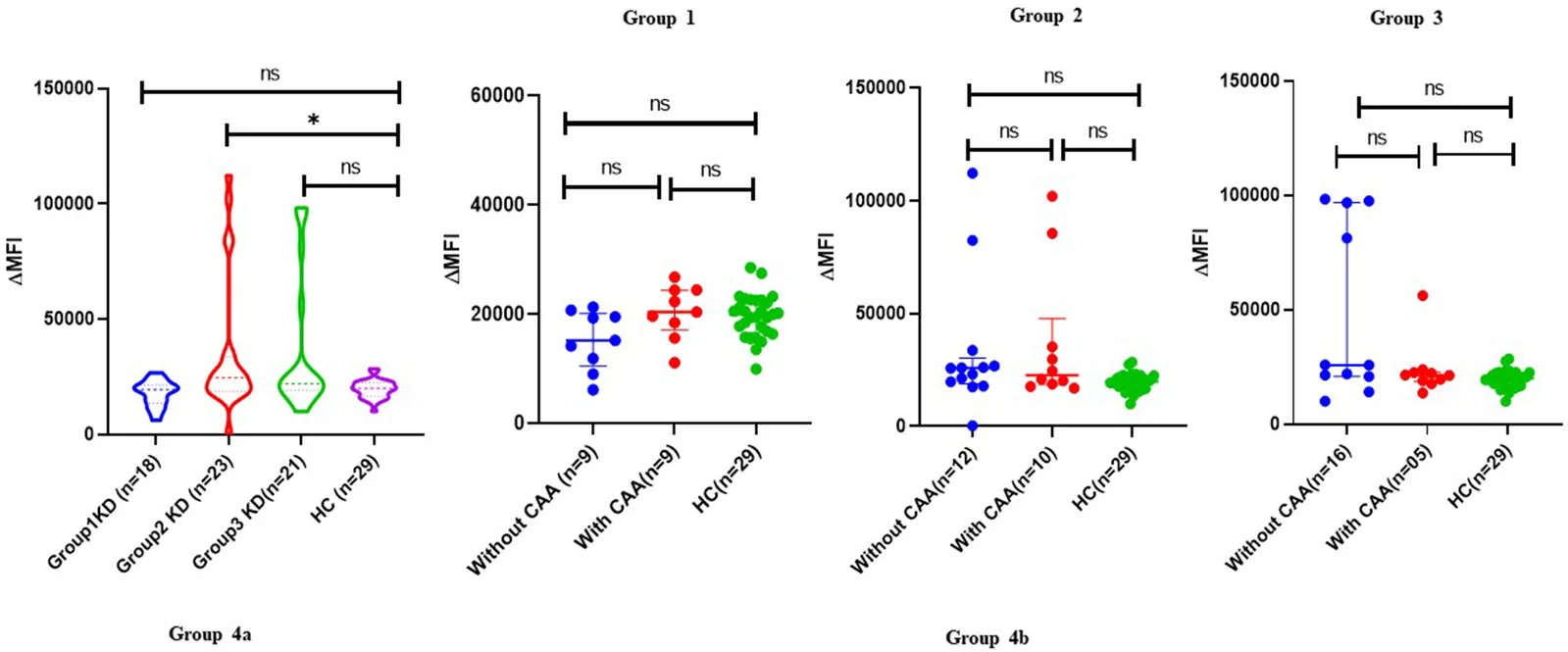
Comparison of ROS production in patients with KD and HC (**a**). Comparison of ΔMFI between KD patients across different study groups and healthy controls (**b**). Subgroup analysis comparing KD patients with and without CAAs and HC. Abbreviations: ROS-reactive oxygen species, KD-Kawasaki disease, HC-healthy control, ΔMFI-delta median fluorescence intensity, CAAs-coronary artery aneurysms

### Inclusion and exclusion criteria for KD patients and healthy controls

Children with a confirmed diagnosis of KD who were under follow-up at the study centre were included [1]. Patients with coexisting cardiovascular disease, hypertension, or abnormal lipid profiles (including inherited lipid disorders) were excluded. Age- and sex-matched healthy children were enrolled as controls. Controls with a history of cardiovascular disease, inherited lipid disorders, diabetes, thrombocytopenia, chronic systemic illnesses (such as renal disease), or haematological malignancies were excluded.

### Sample collection

Three millilitres (3mL) of blood was collected aseptically from patients and HC. Of this, 2 mL was collected in a plain vial for serum separation, and 1 mL was collected in a heparinised vial for ROS estimation. Serum samples were separated, aliquoted, and stored at − 80 °C until further analysis.

### Estimation of extracellular nitric oxide levels

Extracellular nitric oxide was measured using a commercially available Nitric Oxide Assay Kit (EMSNO, Invitrogen™, Thermo Fisher Scientific, USA) according to the manufacturer’s instructions. Serum samples from patients and controls were diluted 1:2 with 1× reagent diluent and filtered through 10,000 molecular weight cut-off (MWCO) filters (88513, Pierce™ Protein Concentrator, PES, Thermo Fisher Scientific, USA) by centrifugation at 15,000 × g for 10 min at room temperature. The resulting filtrate was used for subsequent analysis. Nitrite (2,000 µM) and nitrate (1,000 µM) standards were equilibrated to room temperature, and serial dilutions were prepared to generate final concentrations of 200, 100, 50, 25, 12.5, 6.25, and 3.125 µM for nitrite and 100, 50, 25, 12.5, 6.25, and 3.125 µM for nitrate. The assay was performed as per the standard protocol, and optical density was measured at 540 nm using an Infinite M200 PRO microplate reader (Tecan Group Ltd, Switzerland). Standard curves for nitrite and nitrate were generated by plotting optical density against concentration (µmol/L). Nitrite concentration in the samples was determined directly (X µmol/L), while total nitrite (after nitrate reduction) was measured as Y µmol/L. Nitrate concentration was calculated as the difference between total nitrite and measured sample nitrite, i.e., (Y − X) µmol/L.

### Estimation of ROS in neutrophils by dihydro rhodamine 123 (DHR) assays

ROS production was estimated using dihydro-rhodamine 123, an uncharged, non-fluorescent compound that can diffuse across the cell membrane and be oxidised to rhodamine 123 (a fluorescent compound) by ROS. The resulting fluorescence intensity, proportional to ROS generation, was measured by flow cytometry.

For the assay, 1 mL of heparinised blood from both patients and controls was used. DHR (D1054, Sigma-Aldrich, USA) was added to both unstimulated and stimulated tubes at a working concentration of 7.5 µg/100 µL (stock: 1 mg/100 µL). After a 5-minute incubation at 37 °C in a water bath, phorbol myristate acetate (PMA) (P8139, Sigma Aldrich, USA) was added to the stimulated tube at a working concentration of 2 µg/ml (stock: 1 mg/mL), followed by a 15-minute incubation at 37 °C in the dark. Red blood cells were lysed using 1X RBC lysis buffer (349202, Becton Dickinson, USA), then washed with 1X PBS. Finally, the cells were resuspended in 300 µl of sheath fluid. Samples were analysed on a Navios flow cytometer (Beckman Coulter, USA), and the data were analysed using Kaluza analysis software (Version 2.1). The gating strategy used for ROS measurement in neutrophils is shown in Fig. 2. ROS production was expressed as the difference in median fluorescence intensity (ΔMFI) between stimulated and unstimulated neutrophils: ΔMFI = MFI (stimulated) − MFI (unstimulated).

### Statistical analysis

Results were analysed using GraphPad Prism 9 Software (8.3.0.538) and IBM SPSS Statistics 26.0. Initially, all data types were tested for skewness. After obtaining skewness scores and skewness errors, the data were divided into two groups (skewed and non-skewed). Statistical comparisons among three groups of patients with KD and HC were performed using one-way analysis of variance (ANOVA) followed by the Kruskal-Wallis test for nonparametric variables. Correlations between different parameters were calculated according to Spearman’s correlation coefficient. A value of *p* < 0.05 was considered significant. Graphs were obtained from GraphPad and SPSS.

## Results

A total of 62 patients with KD were included. CAAs were identified in 29 patients (13 transient and 16 persistent), while 33 patients had no evidence of CAAs. Based on Z-score assessment, the left main coronary artery (LMCA) showed small aneurysms (Z score + 2.5 to < + 5) in 1.88% of patients and medium-sized aneurysms (Z score > + 5 to < + 10) in 3.77%, with 94.33% having Z scores < + 2.5. For the left anterior descending artery (LAD), small aneurysms were observed in 5.66% and giant aneurysms (Z score > + 10) in 7.54%, while 86.79% had Z scores < + 2.5. In the right coronary artery (RCA), 5.66% of patients had small aneurysms, 1.88% had medium-sized aneurysms, and 92.45% had Z-scores < + 2.5. For the left circumflex artery (LCx), small, medium-sized, and giant aneurysms were observed in 5.88%, 5.88%, and 5.9% of patients, respectively, and 82.3% had Z-scores < + 2.5.

### Clinical characteristics of the study population

There was a male predominance in the cohort (male-to-female ratio of 3.1:1). The median age at diagnosis was 4 years (range: 0.25 months to 13 years), with 6 patients (9.67%) aged ≥ 10 years. Fever was the presenting feature in all patients. Other clinical manifestations included skin peeling (*N* = 47, 75.80%), oral mucosal erythema (*N* = 50, 80.64%), Beau’s lines (*N* = 22, 35.48%), conjunctival injection (*N* = 32, 51.61%), strawberry tongue (*N* = 36, 58.06%), rash (*N* = 35, 56.45%), swelling of hands and feet (*N* = 23, 37.09%), lymphadenopathy (*N* = 19, 30.64%), and chromonychia (*N* = 11, 17.74%). Reactivation of the BCG vaccination site was observed in 5 patients (8.06%). A positive family history was noted in one case, with an affected sibling. Baseline demographic and clinical characteristics were comparable across the 3 groups, suggesting that these measured variables were unlikely to confound the observed outcomes. Detailed demographic distribution and clinical characteristics are summarized in Supplementary Table S1. Hematological abnormalities at enrollment included thrombocytosis (*N* = 18, 29.03%) and leukocytosis (*N* = 13, 20.96).

### Serum nitrite and nitrate levels in patients with KD and healthy controls

In the intergroup comparison, serum nitrite levels showed limited variation and did not differ significantly between patients with KD (Group 1: 3.46 ± 1.64; Group 2: 5.04 ± 2.55; Group 3: 5.22 ± 3.00) and HC (3.33 ± 1.98) (*p* = 0.06) (Fig 3a). Serum nitrate levels in patients with KD across all three groups (Group 1: 63.07 ± 41.32; Group 2: 58.63 ± 27.12; Group 3: 64.57 ± 26.36) were comparable with HC (57.49 ± 8.68) (*p* = 0.43) (Fig. 3c). Subgroup analysis within the KD cohort was performed to examine the association between nitric oxide metabolites and CAAs. Patients with CAAs showed comparable nitrate levels compared with those without CAAs across all groups (Group 1: *p* = 0.60; Group 2: *p* > 0.99; Group 3: *p* = 0.10) (Fig. 3d). In addition, serum nitrite levels were also comparable between KD patients with and without CAAs (Group 1: *p* > 0.99; Group 2: *p* > 0.99; Group 3: *p* = 0.90) (Fig. 3b).

### Reactive oxygen species production in KD patients and healthy controls

Comparative analysis showed higher ROS production in patients with KD compared with HC. ΔMFI values were higher in Group 2 (35,010 ± 29,660) and Group 3 (35,745 ± 30,132) compared with HC (19,612 ± 3,976) (overall p value was 0.005 when compared between the three groups) while a p- value of 0.03 was achieved when the ΔMFI of ROS was compared between the HC and group 2 KD patients. (Fig. 4a). Intragroup analysis within the KD cohort demonstrated variation in ROS production according to coronary artery status. In Groups 1 and 3, patients with CAAs and those without CAAs had comparable ΔMFI values with statistically nonsignificant differences (*p* = 0.10). In Group 2, ROS production was also comparable between patients with and without CAAs (*p* > 0.99) (Fig. 4b).

### Correlation analysis

Correlation analysis was performed to evaluate the relationship between oxidative stress markers and inflammatory, cardiac, and echocardiographic parameters.

Serum nitrite and nitrate levels showed negative correlations with C-reactive protein (CRP) (*p* = 0.19; *p* = 0.44, respectively) and pro-B-type natriuretic peptide (pro-BNP) (*p* = 0.29; *p* = 0.48), but these associations were not statistically significant. Similarly, ROS showed a weak positive association with CRP (*p* = 0.23) and pro-BNP (*p* = 0.31), although these correlations also did not reach statistical significance. When correlated with coronary artery measurements, Z-scores of LMCA, LAD, and RCA at enrolment showed weak negative correlations with nitrite levels and ROS. In contrast, nitrate levels showed positive correlations with coronary artery Z-scores; however, these associations did not reach statistical significance. Overall, while trends toward associations between oxidative stress markers and inflammatory and cardiac parameters were observed, none of these correlations reached statistical significance (Supplementary Table S2).

## Discussion

Cardiovascular complications remain the most serious consequence of KD, with CAAs developing in up to 25% of untreated patients. This has long-term implications as CAAs increase the risk of cardiovascular sequelae and sudden cardiac death [15]. Early recognition and prompt treatment during the acute phase are therefore essential to prevent irreversible coronary damage.

Although early immunomodulatory therapy has reduced overt coronary involvement, evidence indicates that subclinical ED may persist beyond the acute phase and contribute to long-term vascular morbidity [16]. Previous studies have primarily relied on imaging-based techniques, such as carotid intima–media thickness and flow-mediated dilation, to assess vascular health [4, 17, 11, 18]. While informative, these approaches do not provide insight into the underlying molecular mechanisms of endothelial injury. In clinical practice, KD diagnosis continues to rely on characteristic clinical features and exclusion of similar conditions, with vascular imaging serving as a supportive tool.

In this study, we provide biochemical evidence of persistent oxidative stress and dysregulation of ROS in late-stage KD. Serum nitrate levels in KD patients across all time-stratified groups were comparable with those of HC. Notably, patients with CAAs also exhibited nitrate levels comparable to those without coronary involvement. Although both nitrite and nitrate are metabolites of nitric oxide, our findings indicate that, in contrast to nitrite levels, nitrate may be a more stable and clinically relevant marker of cumulative NO production and oxidative flux if validated in larger cohorts [19]. Previous studies have suggested increased neutrophil NO production during the acute phase of KD [20], with overproduction of NO often indicating an aberrant immune response. Elevated NO levels in KD patients have also been associated with coronary artery dilation [21]. Additionally, previous studies have documented reduced brachial artery FMD in KD patients, indicating persistent abnormal systemic endothelial function even years after the resolution of acute symptoms [4].

Mechanistically, these findings support sustained activation of inducible nitric oxide synthase (iNOS) in the setting of chronic immune activation. Excess NO, in the presence of ROS, is rapidly converted to peroxynitrite, a potent oxidant capable of inducing lipid peroxidation, protein nitration, and endothelial injury. Thus, elevated nitrate levels likely reflect both increased NO synthesis and its downstream oxidative metabolism, linking NO imbalance to vascular damage in KD.

Compared with controls, we observed significantly increased ROS production in KD patients, indicating persistent oxidative stress. Intragroup analysis showed comparable ROS production in all 3 groups between patients with and without CAAs. However, previous studies documented increased oxidative stress in KD patients with coronary aneurysms, showing its association with vascular thickening and stiffening [16].

Neutrophils, a key effector population in KD, generate ROS via NADPH oxidase activation, and our findings suggest that this pro-oxidative phenotype may persist beyond clinical resolution. Although the relationship between ROS levels and CAAs varied across subgroups, further studies with larger cohorts may be required to clarify this association. However, the overall elevation supports a sustained redox imbalance. These findings align with previous studies reporting increased ROS production during the acute phase of KD [22]. For instance, Higashi et al. highlighted the role of oxidative stress in ED [23]. Also, the current significantly elevated ROS levels in patients with KD support previous evidence that OS is a prominent feature that contributes to vascular inflammation and endothelial injury [24].

The present study accounts for discordant findings between ROS and NO metabolites, although both are markers of oxidative stress. The compensatory upregulation of nitric oxide synthase activity during inflammation maintains circulating nitrite and nitrate concentrations within a relatively stable range [25]. Previous studies indicate that enhanced ROS may reduce NO bioavailability through oxidative interactions, such as peroxynitrite formation, thereby contributing to ED (26–27). Also, both ROS and NO are regulated by different biochemical pathways and therefore do not necessarily change in parallel under inflammatory conditions [28]. Consequently, the observed elevation in ROS in the absence of significant differences in NO levels may indicate predominant oxidative stress without substantial disruption of systemic nitrosative homeostasis [25, 28].

We observed a positive correlation between ROS and inflammatory markers (CXCL8, PTX-3, OPN), consistent with findings from prior studies. Dalla et al. reported elevated CRP levels in KD patients with coronary arterial involvement, supporting our finding of a positive correlation between ROS and CRP [29]. Furthermore, Hu et al. identified elevated plasma BNP levels in KD patients, implicating a role for BNP in disease pathology, consistent with our observation of a positive correlation between ROS and Pro-BNP levels [30]. Overall, our study reinforces the notion that oxidative stress persists even after the acute phase of KD.

There is limited long-term follow-up data on KD patients from developing countries. Variations in clinical presentation and outcomes across regions further highlight the need for region-specific data. The present study contributes to this gap by providing biochemical evidence of persistent oxidative stress in a follow-up cohort. From a clinical perspective, persistently elevated ROS levels may reflect ongoing vascular stress and could have potential utility as a biomarker of coronary artery abnormalities and subclinical endothelial injury in KD patients on follow-up. However, these findings are not corroborated for nitrite and nitrate, so further studies with a large sample size are required to determine their clinical relevance.

## Limitations of the study

This study has several limitations. The sample size was relatively small. Patients were stratified by time since diagnosis; however, longitudinal follow-up of the same individuals was not conducted. As a result, comparisons were made between groups rather than within individuals over time. In addition, a broader panel of oxidative stress and endothelial biomarkers could not be evaluated due to resource constraints. Also, subgroup analyses should be considered exploratory and hypothesis-generating, and adequately powered studies are required to confirm these observations. Large prospective longitudinal studies with serial assessments are needed to confirm present findings and to better define temporal changes and their clinical implications.

## Conclusion

The present study demonstrates persistent oxidative stress and elevated ROS levels in patients with KD during long-term follow-up. Elevated ROS levels reflect ongoing immune activation and endothelial injury and may serve as an accessible biomarker of CAAs and long-term cardiovascular risk. This is the first such study from North India to demonstrate an association between persistent oxidative stress markers and CAAs in KD patients at follow-up. Further longitudinal studies in larger cohorts are required to validate these observations and to clarify the potential role of oxidative stress markers in long-term risk assessment in children with KD.

## Electronic supplementary material

Below is the link to the electronic supplementary material.

Supplementary Material 1

Supplementary Material 2

## Data Availability

The data is available upon request.

## Abbreviations

KD: Kawasaki disease
ED: Endothelial dysfunction
CAA: Coronary artery abnormalities
NO: Nitric oxide
ROS: Reactive oxygen species
AHA: American Heart Association
MWCO: Molecular weight cut-off
DHR: Dihydrorhodamine
MFI: Median fluorescence intensity
SI: Staining index
LAD: Left anterior descending
RCA: Right coronary artery
LMCA: Left main coronary artery
ANOVA: Analysis of variance
CRP: C-reactive protein
FMD: Flow-mediated dilation
ROM: Reactive oxygen metabolites
OPN: Osteopontin
PTX-3: Pentraxin-3
CiMT: Carotid intima-media thickness
PMA: Phorbol myristate acetate
