# Supplementary material for: Association of persistent oxidative stress markers with coronary artery abnormalities in Kawasaki disease patients on follow-up
Source: Pediatr Rheumatol Online J. 2026 Sep 4;24:61. doi: 10.1186/s12969-026-01249-w (PMC13563887; doi:10.1186/s12969-026-01249-w)
Supplement: Supplementary file 1 [file 12969_2026_1249_MOESM1_ESM.docx]

| **Clinical characteristics** | **Total KD patients (N=62)** | **Group I**  **(N=19)** | **Group II**  **(N=24)** | **Group III**  **(N=22)** | **p-value** |
| --- | --- | --- | --- | --- | --- |
| **Age** | 4 years (0.25 months to 13 years) | 5.9 years | 4.08 years | 4.34 years | 0.23 |
| **Male: Female** | 3.13 | 4.33 | 2.42 | 3.4 | 0.65 |
| **Fever** | 62(100) | 16 | 24(100) | 21(95.45) | 0.37 |
| **Skin peeling** | 47(75.80) | 9(47) | 22(91.66) | 20(90.90) | 0.61 |
| **Redness of the oral cavity** | 50(80.64) | 13(68.42) | 24(100) | 14(63.63) | 0.45 |
| **Conjunctival injection** | 32(51.61) | 6(31.57) | 12(50) | 13(59.09) | 0.17 |
| **Beau’s line** | 22(35.48) | 5(26.31) | 11(45.83) | 7(31.81) | 0.42 |
| **Strawberry tongue** | 36(58.06) | 6(31.57) | 21(87.5) | 8(36.36) | 0.65 |
| **Rash** | 35(56.45) | 9(47) | 18(75) | 8(36.36) | 0.54 |
| **Swelling of hands and/or feet** | 23(37.09) | 4(21.05) | 11(45.83) | 7(31.81) | 0.22 |
| **Lymphadenopathy** | 19(30.64) | 3(15.78) | 9(37.5) | 7(31.81) | 0.18 |
| **Thrombocytosis** | 18(29.03) | 6(31.57) | 11(45.83) | 7(31.81) | 0.10 |
| **Leucocytosis** | 13(20.96) | 7(36.84) | 17(70.83) | 9(40.9) | 0.38 |
| **BCG site reactivation** | 5(8.06) | 2(10.52) | 1(4.16) | 2(9.09) | 0.002 |

**Supplementary Table 1**
