# Supplementary material for: Association of persistent oxidative stress markers with coronary artery abnormalities in Kawasaki disease patients on follow-up
Source: Pediatr Rheumatol Online J. 2026 Sep 4;24:61. doi: 10.1186/s12969-026-01249-w (PMC13563887; doi:10.1186/s12969-026-01249-w)
Supplement: Supplementary file 2 [file 12969_2026_1249_MOESM2_ESM.docx]

| **Oxidative stress biomarkers** | | | | | | |
| --- | --- | --- | --- | --- | --- | --- |
| **Parameters** | **Nitrite** | | **Nitrate** | | **ROS** | |
|  | **Correlation coefficient** | **p-value** | **Correlation coefficient** | **p-value** | **Correlation coefficient** | **p-value** |
| **CRP (mg/L)** | -0.25 | 0.19 | -0.14 | 0.44 | 0.22 | 0.23 |
| **Pro-BNP (pg/ml)** | -0.21 | 0.29 | 0.14 | 0.48 | 0.19 | 0.31 |
| **ROS (∆MFI)** | 0.08 | 0.51 | -0.05 | 0.65 | - | - |
| **LMCA (Z Score)** | -0.05 | 0.73 | 0.07 | 0.62 | -0.17 | 0.22 |
| **LAD (Z Score)** | -0.13 | 0.35 | 0.17 | 0.23 | -0.14 | 0.3 |
| **RCA (Z Score)** | -0.11 | 0.45 | 0.05 | 0.73 | -0.23 | 0.1 |
| **LCx (Z Score)** | 0.01 | 0.95 | 0.46 | 0.06 | 0.19 | 0.47 |

**Supplementary Table 2**
